# Rouse Up

**Published:** 1873-06

**Authors:** 


### ROUSE UP.

Many a person fails to get well, and sinks into the grave from the want of something to wake up to life.

An old woman had been confined to her bed for many months with a rheumatic affection in one of her limbs. One day, from various causes, all the members of the family had left, and she was alone, with both front and rear doors open. By some freak or other a mad bull came running that way, his tail high in air, his shaggy head shaking ominously, and a roar which sent all the chickens to roost and the old lady out of the back door, and she has never been lame since.

Very recently a young lady who had lost her voice some months ago, was so taken by surprise at the unexpected return of a young friend that she fairly screamed, and her voice became natural immediately. The philosophy of cure is that the desperate efforts forced the blood and nervous energies through the channels which disease had clogged up, and thus restored the natural circulation.

During the war about a hundred men of the Illinois 47th, at Jefferson City, Mo., had been some time shut up in a hospital. Nothing appeared to do them much good; all remedial means seemed to have lost their usual efficacy, and the army surgeons themselves were on the point of despairing of being able to do them any further good, when an order came unexpectedly from headquarters in a great emergency, admitting not a day's delay, no not an hour. "Forward all" was the cry. In a single moment the men seemed to have forgotten that they had ever been sick, and were off for the battle-field. Don't tell the sick that there is nothing the matter with them, nor that they are dangerously ill; make them forget their sickness, wake them up to hope and life again, and all will be well, in many cases. Never allow a patient to fall into despondency and hopelessness. Many times a man is restored to health by giving him something to do which will enable him to make money encouragingly. If any impecunious invalid doubts this, let him try it; it is a remedy which will work to a charm, only if you can get the thing to do.

